# Bicondylar tibial plateau fractures treated with fine-wire circular external fixation

**DOI:** 10.1007/s11751-014-0185-z

**Published:** 2014-02-09

**Authors:** N. Ferreira, L. C. Marais

**Affiliations:** Tumour, Sepsis and Reconstruction Unit, Department of Orthopaedic Surgery, Greys Hospital, University of KwaZulu-Natal, Private Bag X9001, Pietermaritzburg, 3201 South Africa

**Keywords:** Tibial plateau fracture, External fixator, Ilizarov

## Abstract

Bicondylar tibial plateau fractures are serious injuries to a major weight-bearing joint. These injuries are often associated with severe soft tissue injuries that complicate surgical management. We reviewed 54 consecutive patients who sustained bicondylar tibial plateau fractures that were treated with limited open reduction and cannulated screw fixation combined with fine-wire circular external fixation. Forty-six patients met the inclusion criteria of this retrospective review. Eight patients were excluded because they did not complete a minimum of 1-year follow-up. Thirty-six patients had Schatzker type-VI, and ten patients had Schatzker type-V fractures. All fractures were united without loss of reduction; there were no incidences of wound complications, osteomyelitis or septic arthritis. The average Knee Society Clinical Rating Score was 81.6, translating to good clinical results. Minor pin track infection was the most common complication encountered. This review concludes that fine-wire circular external fixation, combined with limited open reduction and cannulated screw fixation, consistently produces good functional results without serious complications.

## Introduction

Bicondylar tibial plateau fractures are serious injuries that are often difficult to treat, even for the experienced trauma surgeon [1–4]. These fractures are frequently the result of high-energy trauma, and the condition of the soft tissues often mirrors the underlying bony injury [4–6]. These injuries are frequently associated with compartment syndrome or may present as open fractures [7].

Tibial plateau fractures are complex injuries that affect a major weight-bearing joint and are associated with significant morbidity [3, 8]. Owing to the viscoelastic properties of bone, the tibia absorbs a large amount of energy at the time of injury. This energy is then expelled into the soft tissue envelope once bony failure occurs. The subcutaneous nature of the proximal tibia results in this energy being absorbed by a very thin soft tissue layer. This soft tissue envelope is intolerant of extensive dissection with implications for surgical management of the underlying bony injury [2, 3, 5].

Open reduction and internal fixation, although convenient for the patient, have been associated with serious complications [5]. Several reports have shown a high incidence of wound complications with possibilities of deep sepsis and chronic osteomyelitis [5, 9–13].

Different strategies have been developed to overcome these complications with varying degrees of success. Monolateral external fixators have managed to decrease the soft tissue complications but not always maintained reduction until union [14, 15]. Circular external fixation has shown the ability to decrease soft tissue complications while providing stable fixation until union [16–19].

This retrospective study reports on the outcome from the management of high-energy tibial plateau fractures through limited open reduction and fine-wire circular external fixation.

## Materials and methods

Fifty-four patients (19 females and 35 males) with high-energy tibial plateau fractures were treated at our tertiary level government hospital between July 2008 and January 2012. All patients were treated with fine-wire circular external fixators for definitive management. The records of the skeletally mature patients with high-energy tibial plateau fractures were reviewed; no patients were lost to follow-up, but eight patients were excluded due to a follow-up period of <1 year. There were 46 patients (17 females and 29 males) who met the inclusion criteria (Table 1).

**Table 1:** Patient demographics

	Age/gender	Mechanism of injury	Schatzker	Soft tissue injury		Additional injuries
				Tscherne	Gustilo–Anderson	
1.	38 M	Pedestrian accident	VI		IIIB	
2.	43 F	Motor vehicle accident	VI	III		
3.	29 M	Pedestrian accident	VI	III		Contralateral tibial plateau fracture
4.	34 M	Motor vehicle accident	VI		IIIA	
5.	40 F	Fall	VI	III		
6.	57 M	Pedestrian accident	VI	III		
7.	59 F	Pedestrian accident	VI	II		Femur fracture
8.	19 M	Pedestrian accident	VI		IIIB	Femur fracture
9.	56 F	Motor vehicle accident	VI	III		
10.	42 M	Fall	VI	III		
11.	37 M	Fall	VI	III		
12.	21 F	Motor vehicle accident	V	III		Contralateral tibia fracture
13.	51 F	Motor vehicle accident	VI	III		Acetabulum fracture, Monteggia fracture
14.	50 M	Motor vehicle accident	VI	III		
15.	40 M	Assault	V	II		
16.	50 M	Motor vehicle accident	V	II		
17.	52 M	Pedestrian accident	VI	III		
18.	26 M	Motor vehicle accident	VI	III		Bilateral femur fracture
19.	27 M	Motor vehicle accident	V	II		
20.	59 M	Motor vehicle accident	VI	III		
21	41 F	Fall	V	III		
22.	46 M	Fall	VI	III		
23.	34 M	Fall	VI	III		
24.	43 F	Fall	VI	III		Clavicle fracture
25.	57 M	Motor vehicle accident	VI	II		Bilateral plateau fracture
26.	57 M	Motor vehicle accident	VI	II		Bilateral plateau fracture
27.	45 M	Motor vehicle accident	VI		IIIA	
28.	36 M	Fall	VI	III		
29.	53 M	Bicycle accident	VI	III		
30.	43 M	Pedestrian accident	VI	III		
31.	24 F	Fall	V	II		
32.	37 M	Motor vehicle accident	V	I		
33.	28 F	Pedestrian accident	VI	II		
34.	33 M	Fall	VI	III		Intercondylar femur fracture
35.	26 F	Fall	V	I		
36.	51 M	Pedestrian accident	VI	III		
37.	52 F	Fall	VI	III		
38.	47 M	Motor vehicle accident	VI		IIIA	
39.	41 M	Fall	V	I		
40.	52 F	Fall	VI	III		
41.	52 M	Pedestrian accident	VI	III		
42.	68 F	Motor vehicle accident	VI	III		
43.	37 F	Fall	V	III		
44.	56 F	Fall	VI	III		
45.	48 M	Motor vehicle accident	VI	III		Femur, tibia, tibial pilon fracture
46.	41 F	Pedestrian accident	VI	II		

The mean age was 43 years (SD 11.1, range 19–68 years). Mechanisms of injury included pedestrian-vehicle accidents (*n* = 12), occupants in motor vehicle accidents (*n* = 17), falls from height (*n* = 16), and assaults (*n* = 1). Ten patients also required treatment for associated musculoskeletal injuries (Table 1).

**Fig. 1:**
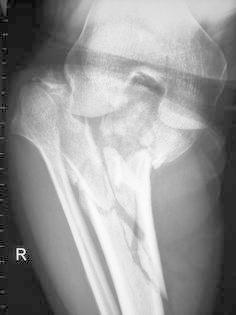
52-year-old female with bicondylar tibial plateau fracture

Host staging for comorbid issues included systemic factors such as HIV infection and CD_4_ count as well as any chronic medical conditions. HIV infection was found in seven patients (15 %) with CD_4_ counts ranging from 309 to 612 cells/mm^3^ (median = 352 cells/mm^3^, SD 104.9). There were two diabetic patients, and six patients were smokers (Table 2). Local staging included classifying the soft tissue injuries according to the Gustilo–Anderson classification for open fractures and the Tscherne and Goetzen classification for closed fractures [20, 21]. There were plain X-rays and CT scans for all patients (Fig. 1). Fractures were classified according to the Schatzker classification [22]. Most of the patients sustained Schatzker type-VI fractures (*n* = 36) with the remainder being Schatzker type-V fractures (*n* = 10).

**Table 2:** Management and complications

	Co-morbidities	Fixator	Time to union (weeks)	Complications	Follow-up (months)
1.	None	Ilizarov	34	Meta-diaphyseal non-union	18
2.	None	Ilizarov	14	None	24
3.	None	Ilizarov	18	None	24
4.	None	Ilizarov	20	Compartment syndrome	15
5.	None	Ilizarov	16	Pin tract sepsis C&O II	12
6.	None	Ilizarov	17	Pin tract sepsis C&O II	12
7.	None	Ilizarov	28	None	12
8.	None	Ilizarov	22	None	21
9.	None	Ilizarov	26	Pin tract sepsis C&O II	13
10.	None	Ilizarov	20	Pin tract sepsis C&O II	12
11.	None	Ilizarov	20	None	12
12.	None	Ilizarov	16	None	13
13.	None	Ilizarov	19	None	14
14.	None	Ilizarov	16	None	12
15.	None	Truelok	14	Pin tract sepsis C&O II	13
16.	Smoker	Ilizarov	12	Pin tract sepsis C&O IV	12
17.	None	Ilizarov	18	None	17
18.	HIV positive (CD_4_ = 360)	Truelok	54	Meta-diaphyseal delayed union	25
19.	None	Ilizarov	16	None	15
20.	None	Ilizarov	10	None	13
21	None	Truelok	21	None	14
22.	HIV positive (CD_4_ = 352)	Ilizarov	20	None	12
23.	None	Ilizarov	13	Compartment syndrome	24
24.	None	Ilizarov	19	None	15
25.	Diabetes mellitus	Truelok	13	None	24
26.	Diabetes mellitus	Truelok	13	None	24
27.	Smoker	Ilizarov	19	Pin tract sepsis C&O III	15
28.	HIV positive (CD_4_ = 309)	Ilizarov	15	None	14
29.	None	Truelok	18	Pin tract sepsis C&O II	18
30.	None	Truelok	17	None	12
31.	None	Ilizarov	19	None	12
32.	None	Ilizarov	18	None	13
33.	RVD positive (CD_4_ = 314)	Truelok	15	None	14
34.	None	Truelok	16	None	13
35.	None	Ilizarov	9	None	12
36.	HIV positive (CD_4_ = 612)	Truelok	10	None	17
37.	Smoker	Truelok	16	Pin tract sepsis C&O II	13
38.	None	Truelok	16	None	12
39.	Smoker	Ilizarov	19	Pin tract sepsis C&O II	12
40.	Smoker	Ilizarov	17	Pin tract sepsis C&O II	12
41.	None	Truelok	16	None	13
42.	None	Truelok	14	None	12
43.	HIV positive (CD_4_ = 407)	Truelok	16	None	13
44.	None	Truelok	15	None	13
45.	HIV positive (CD_4_ = 347)	Ilizarov	21	None	14
46.	Smoker	Ilizarov	30	None	12

Open fractures occurred in five patients (4 Gustilo–Anderson type IIIA and 1 Gustilo–Anderson type IIIB). These patients were taken to surgery on admission for debridement and temporary joint-spanning monolateral external fixation. Definitive wound cover was performed at 48 h for all open fractures. All Gustilo–Anderson type IIIA wounds were closed with delayed primary closure. A random fasciocutaneous flap was used to close the Gustilo–Anderson type IIIB wound. Compartment syndrome was diagnosed in two patients. Both patients were treated with emergency fasciotomies and temporary joint-spanning monolateral external fixators. Split skin grafts were used to close the fasciotomy wounds in both. The majority of the other patients had significant closed soft tissue injuries; these patients were initially treated with above-knee plaster of Paris backslabs and admitted to the ward for elevation.

The definitive surgical procedure was performed after the CT scan was available. The mean interval between admission and circular fixator application was 8.5 days (median = 7 days, SD 5.5, range 1–21). The condition of the soft tissue envelope did not influence the timing of definitive surgery.

## Surgical technique

**Fig. 2:**
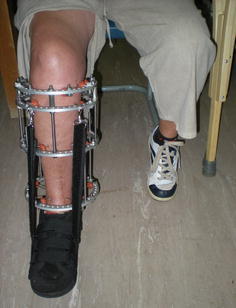
Four ring frame designing with spread along entire length of tibia

A four-ring frame design was used in all cases. It consisted of three full rings and a 2/3rd proximal ring, open posteriorly, to allow knee flexion during rehabilitation. The 2/3rd ring was attached to a full ring via three 30-mm spacers to prevent ring deformation during wire tensioning. The two distal rings were spread along the tibial diaphysis to provide optimal stability, while avoiding a span of >150 mm between any rings (Fig. 2).

The surgical technique included closed reduction or limited open reduction through a 2-cm longitudinal midline incision. Fluoroscopy-guided reduction of the articular surface was performed via this incision and of any meta-diaphyseal fracture lines. If fracture lines were not present or inadequate to allow insertion of instruments for reduction in articular fragments, a cortical window was created. After articular surface elevation, the resulting metaphyseal defect was grafted with iliac crest autograft. Due to the extensive articular comminution that was sometimes seen, anatomical joint reduction was not always possible.

**Fig. 3:**
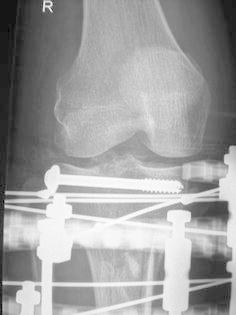
Post-operative radiograph showing limited internal fixation and circular external fixator

After joint line alignment was achieved, a single 6.5-mm cannulated screw was inserted from lateral to medial along the subchondral bone. This resulted in a single articular block and a diaphyseal fragment that could be aligned in the coronal and sagittal planes (Fig. 3). Four 1.8-mm olive wires were placed on the proximal ring, two transverse and two oblique. Two 1.8-mm wires were placed on each of the two distal rings. All wires were tensioned to between 110 and 130 kg. Ilizarov (Smith and Nephew, Memphis, TN) fixators were used in 30 cases and TrueLok (Orthofix, Verona, Italy) fixators in 16 cases.

## Rehabilitation

Post-operative rehabilitation commenced on day one after definitive fixation, and where possible, active-assisted knee range of motion exercises commenced. An attempt to achieve a 90° arc of motion (0°–90°) was made for all patients prior to discharge. Two patients had the initial circular fixators extended across the knee to allow healing of the soft tissues prior to knee mobilization. Once the soft tissues had healed sufficiently, the across-knee extension was removed and active-assisted knee range-of-motion exercises was commenced. All patients were encouraged to mobilize with full weight bearing as comfort allowed. In practice, most patients mobilized partial weight bearing with crutches and progressed to full weight bearing over the ensuing 2–3 weeks. Particular attention was paid to functional rehabilitation and normalization of gait pattern in order to promote recovery and bony union.

Pin site dressings were left undisturbed for the first 7–10 days following the definitive procedure. After this period, the dressings were removed, and twice daily pin site cleaning was commenced. Cleaning was performed in an atraumatic manner with the use of a swab soaked in an alcoholic solution of chlorhexidine. Pin site infections, when they occurred, were graded and treated according to the Checketts and Otterburn classification [23].

All patients were followed up at our orthopaedic outpatient department clinic. Initial 2-weekly follow-up visits were scheduled. The intervals were extended to 4-weekly once robust rehabilitation, and pin track care routines were established. Progress with rehabilitation, follow-up radiographs, and complications were documented with each clinic visit. The average follow-up time was 14.9 months (median = 13 months, SD 4.1, range 12–25). The functional outcome was calculated with the Knee Society Clinical Rating Score [24]. The reported scores were obtained at a minimum follow-up of 6 months after frame removal.

## Results

All fractures united in a mean of 18.3 weeks (median = 17 weeks, SD 7.1, range 9–54; Table 2). Union was assessed clinically and radiologically. Once radiological union was deemed sufficient, the external fixator was dynamized and the patient encouraged to bear weight further. The external fixator was removed once painless weight bearing on a dynamized external fixator was achieved.

There was no loss of fracture reduction. Two patients had delayed union of the metaphyseo-diaphyseal fracture line. One of these patients underwent autologous bone grafting, and union was achieved at 24 weeks. The second patient declined bone grafting and eventually united at 54 weeks without any further surgical intervention.

The Knee Society Clinical Rating Scores ranged from 48 to 100, translating into an average Knee Society Score of 81.6 (median = 83.8, SD 12.9; Table 3). According to the rating, 22 outcomes were excellent, 16 were good, four were fair, and four were poor. The average arc of knee motion was 100.5°, ranging from 35° to 125°. Seven patients developed varying degrees of knee flexion contractures. Three patients had a 5° flexion contracture, while another three had approximately 10° of contracture. A severe flexion contracture of 20° was seen in one patient. Thirty-two patients were able to ambulate without additional support, while 12 patients required a cane and two patients required crutches to aid mobilization.

**Table 3:** Knee Society Clinical Rating Scale

	Knee score	Functional score	Rating scale	Grade
1.	85	90	87.5	Excellent
2.	65	55	60	Fair
3.	94	100	97	Excellent
4.	83	85	84	Good
5.	90	90	90	Excellent
6.	81	100	91	Excellent
7.	88	75	81.5	Good
8.	100	100	100	Excellent
9.	78	55	66.5	Fair
10.	90	90	90	Excellent
11.	80	45	62.5	Fair
12.	99	90	94.5	Excellent
13.	52	55	53.5	Poor
14.	73	75	74	Good
15.	80	85	82.5	Good
16.	78	70	74	Good
17.	88	70	79	Good
18.	78	70	74	Good
19.	95	100	97.5	Excellent
20.	77	80	78.5	Good
21	69	45	57	Poor
22.	92	80	86	Excellent
23.	95	90	92.5	Excellent
24.	93	90	91.5	Excellent
25.	97	100	98.5	Excellent
26.	99	100	99.5	Excellent
27.	75	75	75	Good
28.	83	80	81.5	Good
29.	89	90	89.5	Excellent
30.	90	80	85	Excellent
31.	94	100	97	Excellent
32.	75	80	77.5	Good
33.	87	70	78.5	Good
34.	83	84	83.5	Good
35.	92	70	80.5	Good
36.	97	90	93.5	Excellent
37.	100	72	86	Excellent
38.	94	100	97	Excellent
39.	83	60	71.5	Good
40.	90	90	90	Excellent
41.	68	50	59	Poor
42.	99	75	87	Excellent
43.	90	80	85	Excellent
44.	80	80	80	Good
45.	67	30	48.5	Poor
46.	73	55	64	Fair

Pin site infection was the most common complication occurring in 11 patients (23.9 %). Minor pin site infections occurred in ten of the 11 patients. These included nine Checketts and Otterburn grade II infections which were successfully treated with local pin track care and oral antibiotics. One patient developed a grade III infection which resolved after removal of the offending wire. A major infection occurred in one patient; this Checketts and Otterburn grade IV infection occurred at the end of the treatment period. The frame was removed without compromising the fracture treatment. No patients developed soft tissue complications. Wound dehiscence, infection, osteomyelitis of the fracture, or septic arthritis did not occur.

## Discussion

The surgical treatment of bicondylar tibial plateau fractures is demanding, and the ideal treatment modality remains to be established [3]. In order to minimize joint stiffness and post-traumatic osteoarthritis, early joint mobilization is essential [3, 25, 26]. This is only possible if stable fixation is achieved to allow mobilization without loss of reduction.

In terms of mechanical stability, dual plating has traditionally been considered as the gold standard, as it addresses both the medial and lateral columns [22]. Extensive soft tissue dissection is required to place these plates, and high complication rates have been described, both with single and two incisional approaches [5, 9–13, 27]. Infection rates after open reduction and internal fixation reported by Jiang et al. [13], Yang et al. [27], and Moore et al. [9] are 7.3, 13.6, and 19 % respectively, while Young et al. [10] reported an 87.5 % infection rate after dual plating of high-energy bicondylar tibial plateau fractures. These post-operative infections impart significant additional morbidity. Barei et al. reported an average of 3.3 additional surgical procedures, and Young et al. an average of five additional surgical procedures to treat the infections [5, 10].

In order to minimize the soft tissue dissection in treating such fractures, monolateral external fixators were introduced to provide fracture stabilization. Although this strategy showed a decrease in the soft tissue complications [14, 15], mechanical stability was inadequate and loss of reduction was seen [15]. The reason for this mechanical failure is the cantilever loading forces that the external fixator is subjected to. This places the external fixator at a mechanical disadvantage when having to provide stability at a distance from the fixator body [28].

Circular external fixators utilize beam loading of the tensioned fine wires to provide stability across the entire length of the wire [28]. The surgeon can exploit this property with the use of multiple wires, to provide uniform support for the tibial plateau articular surface that may be likened to a custom raft-construct tailored to each individual fracture pattern. Biomechanical analysis demonstrated that four tensioned olive wires combined with a single lag screw provides better stability than dual plating [29]. For this reason, we always combine a single lag screw with four tensioned olive wires to the proximal ring. This method of fixation provides adequate stability to allow early joint mobilization and weight bearing without risking loss of reduction. None of our patients experienced post-operative loss of reduction when comparing immediate post-operative and latest follow-up radiographs.

An additional advantage with fine-wire circular fixators is the minimally invasive nature of its application [30]. In an area where the soft tissues are often compromised, additional surgical trauma could have undesirable consequences [28, 30]. Fine wires passed through compromised tissues impart almost no additional trauma and do not appear to cause any morbidity. None of our patients had soft tissue complications from either the wire insertion sites or other minimal invasive incision sites. Multiple previous investigations have shown benefit from this surgical technique. Kataria et al. [16] reported on a series of 38 patients and had no incidences of non-union or septic arthritis. Dendrinos et al. [19], Ali et al. [17], and Chin et al. [18] treated 24, 20, and 18 patients respectively, all with no infective complications. Our results add to this body of literature, with no cases of wound dehiscence, infection, osteomyelitis, or septic arthritis encountered.

The benefit of early definitive surgical stabilization is early mobilization. This is advantageous as it decreases delays in functional rehabilitation that may negatively impact outcome. The average functional outcome, measured with the Knee Society Clinical Rating Score achieved in our series, was 81.0. This compares favourably to other published studies with average Knee Rating scores ranging from 65.9 to 80.2 [18, 31–33]. These initial functional results appear to be maintained over the medium-to-long term, as illustrated by Katsenis et al. [34] over a minimum 5-year follow-up.

Pin track sepsis remains a common complication with the use of external fixators [35, 36]. Quoted incidences range from 11.3 to 100 % [37–41]. Fortunately, the majority of these infections are minor and easily treated with local pin track care and oral antibiotics [42]. Intra-operative pin insertion methods that emphasize low energy insertion and a standardized post-operative pin track care protocol have been shown to be effective in reducing the incidence and severity of pin tract sepsis [43]. These strategies should be instituted for all patients who undergo external fixator application. In our series, only one patient developed a major pin track infection that required removal of the external fixator. Ten patients had minor pin track sepsis that responded to local treatment and oral antibiotics. It is interesting to note that HIV infection had no influence on the incidence or severity of pin track sepsis, while five out of six patients who smoked developed pin track sepsis.

## Conclusion

Fine-wire circular external fixation with limited open reduction is an effective treatment for high-energy tibial plateau fractures. This treatment strategy has the ability to produce good functional results for the majority of patients while minimizing serious complications.
